# 115 Pediatric Laser Resurfacing in 579 Patients: Efficacy Analysis of a Multimodal Perioperative Pain Regimen

**DOI:** 10.1093/jbcr/iraf019.115

**Published:** 2025-04-01

**Authors:** Mark Talon, Alexis McQuitty, Ramon Zapata-Sirvent

**Affiliations:** Shriners Children’s - Galveston; Blocker Burn Unit; University of Texas Medical Branch

## Abstract

**Introduction:**

Carbon dioxide (CO2) laser scar resurfacing is the most common surgery performed in our pediatric burn center. It positively impacts a burn patient’s quality of life by reducing scar thickness and pruritis, improving mobility in those with contractures, and improving texture and erythema. Pain associated with large TBSA has been compared to a moderate to severe sunburn and may be poorly tolerated in children. There is limited published data showing the incidence and quality of perioperative pain in these surgeries.

**Methods:**

An IRB exemption was obtained prior to data collection. We performed a chart review for the past 2 years of CO2 laser scar resurfacing to analyze outcomes and the effectiveness of our analgesic program. 539 patients met this criteria, with most receiving therapy for >20% TBSA resurfacing. The age range was between 2 and 20 with a median age of 11 and a SD of 4.56. The average length of surgery was 48.72 minutes and a median of 37 minutes with a SD of 33.59. Our premedication regimen for all patients was the following: 1) < 10 years old--oral midazolam and intranasal dexmedetomidine and 2) >10 yrs old and >30 kg--oral methadone and acetaminophen. In addition, all patients received intraoperative ondansetron, ketorolac, and dexamethasone. Almost 50% of patients, predominantly those < 10 yrs old, received a small dose of intraoperative fentanyl or morphine. Lidocaine/prilocaine cream and ice were applied topically immediately prior to the anesthetic emergence. No opioid prescriptions were provided for postoperative outpatient care.

**Results:**

The average pain score in the immediate postoperative period for all groups prior to discharge was 0.10 with a SD of.48 on a pain scale of 1-10. There were 3 recorded incidents of emesis. There were no post-operative readmissions or respiratory complications.

**Conclusions:**

Laser scar resurfacing surgery, especially for large TBSA, is associated with mild to moderate pain in the pediatric population. Our multimodal regimen of preemptive analgesia and antiemetics appears to be effective in managing pain and preventing postoperative emesis. Patients do not require additional outpatient opioids. A limitation of this study is the retrospective analysis of the data. Future goals include limiting or avoiding intraoperative opioids.

**Applicability of Research to Practice:**

This retrospective analysis of our current patient care regimen is effective and avoids the need for outpatient opioid use. Multimodal management of pain and nausea also improves patient satisfaction overall and has a high safety profile.

**Funding for the Study:**

N/A

